# Assessing the Effects of Multiple Stressors on the Recruitment of Fruit Harvested Trees in a Tropical Dry Forest, Western Ghats, India

**DOI:** 10.1371/journal.pone.0119634

**Published:** 2015-03-17

**Authors:** Anita Varghese, Tamara Ticktin, Lisa Mandle, Snehlata Nath

**Affiliations:** 1 Keystone Foundation, P.B. 35, Kotagiri, The Nilgiris 643217 India; 2 Botany Department, University of Hawaii at Manoa, 3190 Maile Way, Honolulu, Hawaii 96822, United States of America; 3 Natural Capital Project, Stanford University, 371 Serra Mall, Stanford, CA 94305, United States of America

## Abstract

The harvest of non-timber forest products (NTFPs), together with other sources of anthropogenic disturbance, impact plant populations greatly. Despite this, conservation research on NTFPs typically focuses on harvest alone, ignoring possible confounding effects of other anthropogenic and ecological factors. Disentangling anthropogenic disturbances is critical in regions such as India’s Western Ghats, a biodiversity hotspot with high human density. Identifying strategies that permit both use and conservation of resources is essential to preserving biodiversity while meeting local needs. We assessed the effects of NTFP harvesting (fruit harvest from canopy and lopping of branches for fruit) in combination with other common anthropogenic disturbances (cattle grazing, fire frequency and distance from village), in order to identify which stressors have greater effects on recruitment of three tropical dry forest fruit tree species. Specifically, we assessed the structure of 54 populations of *Phyllanthus emblica*, *P*. *indofischeri* and *Terminalia chebula* spread across the Nilgiri Biosphere Reserve, Western Ghats to ask: (1) How are populations recruiting? and (2) What anthropogenic disturbance and environmental factors, specifically forest type and elevation, are the most important predictors of recruitment status? We combined participatory research with an information-theoretic model-averaging approach to determine which factors most affect population structure and recruitment status. Our models illustrate that for *T*. *chebula*, high fire frequency and high fruit harvest intensity decreased the proportion of saplings, while lopping branches or stems to obtain fruit increased it. For *Phyllanthus* spp, recruitment was significantly lower in plots with more frequent fire. Indices of recruitment of both species were significantly higher for plots in more open-canopy environments of savanna woodlands than in dry forests. Our research illustrates an approach for identifying which factors are most important in limiting recruitment of NTFP populations and other plant species that may be in decline, in order to design effective management strategies.

## Introduction

Tropical forests provide a number of products and services which form an important part of the lives of human beings that live in close proximity to these areas [1]. Ninety percent of tropical forests lie outside of protected areas [2] and many protected areas are also used to some extent by the millions of forest-dependent people who live in and around them. The harvest of many non-timber forest products (NTFPs) such as fruit, bark, roots, and resins are a cause of high pressure on plant populations in many tropical forests [3]. These products are harvested for food, medicines, and handicrafts, among other uses, both for subsistence and for sale. Harvesting of NTFPs rarely occurs alone, and communities that harvest NTFPs also often graze their livestock on the forest understory, hunt for bush meat, and harvest firewood [4]. As is common across parts of Asia and Africa, many forest-dependent communities also set low-intensity ground fires in the dry season to increase visibility for hunting, stimulate resprouting of grasses for cattle, keep tick populations in check, control invasive species (that may compete with NTFPs or fodder for cattle), and clear pathways to facilitate movement [5]. As a result, populations of NTFP species, including those inside of protected areas, are often subject to multiple concurrent anthropogenic disturbances.

Most studies that have assessed the conservation status or harvest sustainability of NTFP populations have focused exclusively on evaluating the effects of harvesting activity [6]. Harvest of NTFP may be confounded with other anthropogenic disturbances and its effects on regeneration or recruitment, overestimated or underestimated. For example, fire and grazing can mediate the effects of leaf harvest on an understory palm [7]. Targeting the wrong drivers of decline, and/or ignoring other key drivers can lead to ineffective management. In addition, most NTFP studies have assessed the effects of harvest on populations within one site or area [8, 6], and used this to derive recommendations for much larger areas. If the nature and intensity of other types of anthropogenic disturbances vary across sites, this could also lead to inappropriate management recommendations. Disentangling which forms of anthropogenic disturbances are responsible for plant population decline is critical in regions such as India’s Western Ghats, a biodiversity hotspot with very high human density [9]. Therefore identifying strategies that can permit both use and conservation of resources especially in these human dominated landscapes is very important.

We identify drivers of decline for three tropical dry forest trees that represent some of India’s most heavily harvested NTFPs—*Phyllanthus emblica* L. and *P*. *indofischeri* Bennet (Euphorbiaceae), and *Terminalia chebula* Retz. (Combretaceae). The two *Phyllanthus* species are commonly called Indian gooseberry, *amla*, or *nellikai*, while *T*. *chebula* is known locally as *hirda* or *kadukai*. The fruit of these three species are rich in alkaloids and tannins. They are used extensively in pickles, jams, hair dye, shampoos, and cosmetics and cater to the multibillion dollar herbal industry [10]. Harvest of these fruits form an important seasonal livelihood activity of the indigenous people.

Like many Indian NTFPs, *P*. *emblica*, *P*. *indofischeri*. *and T*. *chebula* have been used for millennia [11] but have become heavily commercially harvested in recent decades. The forests from which they are harvested are also subject to multiple other sources of anthropogenic disturbance, including fire, firewood collection, and cattle grazing. In 2000 by an interim order of the Supreme Court of India, the removal of products from a National Park or Sanctuary was declared illegal. The intention was to improve habitat for wildlife, but it was not based on ecological studies of the impacts or harvest of other disturbances, and had large negative economic repercussions for some indigenous communities who depended on NTFP harvest [12]. Research elsewhere in India has illustrated reduced recruitment of *P*. *emblica* and other NTFPs across a gradient of increasing human disturbance [13, 14] but did not determine which of multiple potential disturbance factors were actually responsible for any decline. More recently, mistletoe (*Loranthes* spp.) and invasive species have been shown to be drivers of decline of *P*. *emblica* and *P*. *indofischeri* populations in an Indian protected area [15]. Effective management of these and other species clearly requires identification as to whether they are actually at risk and which types of anthropogenic disturbance or other factors are likely responsible for their decline.

We assessed the population structure of *P*. *emblica*, *P*. *indofischeri* and *T*. *chebula* in 41 one hectare plots that spanned approximately1500 km^2^ of the Nilgiri Biosphere Reserve (NBR) in South India. The NBR is part of the Western Ghats Biodiversity hotspot [16] and is inhabited by about 20 indigenous (*adivasi*) groups. We asked the following questions: 1) How are populations (individuals in the same plot) of the three species recruiting? We used two measures to estimate this: (i) the proportion of saplings (1–5 cm diameter at breast height) and (ii) the coefficient of skewness, a measure of the relative proportion of small versus large individuals. These have been shown to be a reasonable predictor of the direction of population change in other tropical Asian tree species [17]. 2) What types of anthropogenic disturbance, specifically intensity of fruit harvest, lopping branches for fruit harvest, cattle grazing, fire frequency, distance from village (as a proxy for firewood collection and anthropogenic disturbance in general) and environmental factors, specifically mistletoe infection, canopy openness, forest type and elevation are the most important predictors of the recruitment status of these species?

To address our questions we used (1) a participatory approach to involve local communities in the research and triangulate their knowledge of historic and recent disturbance levels with our field observations; and (2) an information-theoretic model-averaging approach to determine the impact and relative importance of multiple anthropogenic disturbances and environmental factors. The combination of these approaches allowed us to obtain robust rankings for a relatively large number of factors that are potentially significant and to identify which of these are most limiting to regeneration in these coupled human-natural systems. We hypothesized that cattle grazing and fire frequency would have a greater effect than fruit harvest on limiting recruitment of the three study species, given their strong potential effects on woody species recruitment in tropical dry forests [18, 19] and the small reported effects of tree fruit harvest for many species [8, 20, 15].

## Methods

### Study Site

The Nilgiri Biosphere Reserve (10º 45’N to 12 º N and 76º E to 77º 5’ E) spans an area of 5520 km^2^ in the Western Ghats mountain chain, and crosses through three South Indian states of Karnataka, Kerala and Tamil Nadu. There are several protected areas within the NBR and still large tracts of forests, known as reserve forests, lie outside of these and are managed for multiple uses. This study took place exclusively in the NBR’s reserve forests.

Vegetation in the NBR ranges from thorn forests to wet evergreen forests, but this study took place in the dry forests, including dry deciduous forests and savanna woodlands [21], which ranged from 600–1500m in elevation. Dry deciduous forests receive between 1,000 and 1,500 mm/yr rainfall and canopy cover is between 40 and 60% [19]. Common tree species include *Terminalia spp*., *Anogeissus latifolia* and *Pterocarpus marsupium*. Savanna woodlands tend to be drier, have more grasses and more open canopies than dry deciduous forests. The most common tree species in the savanna woodlands include *Chloroxylon swietenia*, *Bridelia crenulata*, and *Dodonaea* spp.

This wealth of biological diversity at the NBR is matched by the diversity of indigenous communities [22] that collect NTFPs from reserve forests. NTFPs contribute up to 40% of household income in this region [23]. The indigenous people also use the reserve forests, to graze cattle (which browse the forest understory), collect firewood, as sacred sites and burial grounds. Firewood collection involves the removal of twigs and dead and fallen wood; live wood was not observed to be cut down and there was no commercial market for firewood in the study region. Subsistence agriculture is practiced outside of the reserve forests and within village lands, the main crops are millet, maize, and beans, complemented by plantations of cash crops for coffee and tea. Logging for timber has been prohibited in all forests of the NBR for over 30 years and has been strictly enforced.

### Study Species

*Phyllanthus emblica* and *P*. *indofischeri* are shade-tolerant small trees, growing up to 10 or 15 m in height, respectively. *Phllanthus emblica* is distributed throughout dry deciduous forests of south and Southeast Asia, but *P*. *indofischeri* is endemic to dry forests of South India’s Deccan region [24]. Fruits of both species are 6-seeded berries with fleshy exocarps that mature from December to February. The stony endocarp then dehisces and the seeds are mechanically dispersed. Ungulates eat the fruit and regurgitate the endocarps containing the seeds [25]. *Terminalia chebula* is distributed across the dry forests of peninsular India [26]. It is a shade- tolerant tree that grows up to about 15 m in height. *T*. *chebula* fruit are ridged drupes that mature during September to October.

All three species produce a high abundance of fruit, although inter-annual variation can be high in *Phyllanthus* spp. [27,15]. Individual trees produce fruit once they reach approximately 5 cm diameter at breast height (dbh) for *P*. *indofischeri* and 9–10 cm for *P*. *emblica* and *T*. *chebula* [15]. All three species tend to recruit each year but can be slow-growing, and individuals may take 5–10 years to reach sapling size (Ticktin and Ganesan, unpublished data). Seedlings and saplings of all three species can resprout after low intensity fire.

### Assessment of population structure

**Fig 1:**
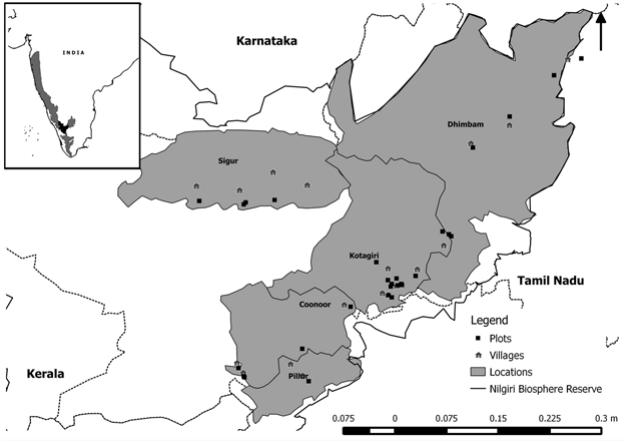
Location of study sites in the Nilgiri Biosphere Reserve, South India. The 1 ha plots were located within reserve forests adjoining harvester villages.

To identify populations of the three study species, we carried out participatory resource appraisal exercises in 30 villages, spanning three different indigenous groups, the Irula, Kurumba and Jenu Kurumba, in five locations where NTFPs are harvested within the NBR (Table 1, Fig. 1) from 2002–2007. Permission to conduct this study in the reserve forests was granted to the Keystone Foundation by the Tamil Nadu Forest Department (Ref. No. RE2/5343/01 dt 17.5.02) and formed a part of the activities of the NTFP project in Keystone in which S.N. and A.V. were actively involved. Oral informed consent was obtained from each village to carry out the research and then individually, prior to each interview with a villager, because some interviewees lacked reading or writing skills. In these exercises, harvesters provided information on the species of NTFP harvested and marked the location of harvesting areas, and forest boundary limits of each village. We then carried out a minimum of two reconnaissance surveys in each village in which we were always accompanied by harvesters from the village. We then established randomly placed 1 ha plots (which were at least 500m apart) in each forest stand that was indicated in the participatory maps as an important area of harvest. Each village had more than one important area of harvest and in many cases these areas were shared with neighboring villages.

**Table 1:** Location, species, and tenural status of NTFP in study plots in the Nilgiri Biosphere Reserve (NBR).

Location in NBR	# NTFP harvester villages surveyed	Commercially harvested NTFP study species	Tenural status of NTFP collection in the region (at the time of the survey)
**Coonoor**	9	*Phyllanthus emblica, Terminalia chebula*	NTFP collection, sale and storage organized through contractors and traders.
**Dhimbham**	5	*Phyllanthus emblica, P. indofischeri, Terminalia chebula*	NTFP collection, sale and storage organized through the local Village Forest Protection Council and the state Forest Department.
**Kotagiri**	7	*Phyllanthus emblica, P. indofischeri, Terminalia chebula,*	The north eastern slopes of the Kotagiri region were closed for NTFP collection on a commercial scale as of four years before this study. The Konavakarai slopes and other forest areas of Kotagiri were open for collection but little occurred, since the option for wage labor in the area was high.
**Pillur**	4	*Phyllanthus emblica, P. indofischeri, Terminalia chebula*	NTFP collection is organized through the Village Forest Protection Councils (VFPC) and the local Self Help Groups (SHG) who take the help of private contractors and traders.
**Sigur**	5	*Phyllanthus emblica*	The area was closed for NTFP collection as of five years before this study.

In each plot we recorded evidence of grazing, firewood collection, fire, and invasive species (Table 2). All individuals (including all visible seedlings <130 cm tall) of the study species were counted, and individuals > 130 cm tall were measured for dbh. The number of individuals per plot ranged from 3–66 for *T*. *chebula* and 11–437 for *Phyllanthus spp*. All plots contained fruit producing trees. In total, we measured 4,307 individuals in 41 plots. We considered the individuals in each plot as separate populations. Since some plots contained multiple study species, we measured a total of 54 populations.

**Table 2:** Classification of disturbance types assessed as predictors of regeneration status of non-timber forest products (NTFPs) harvested in the Nilgiri Biosphere Reserve.

Type of disturbance	Low	Medium	High
**Fruit harvest level**	On average <10% of trees harvested annually for fruit. Subsistence NTFP collection taking place.	On average 20–40% of trees harvested annually for fruit. NTFP collection for commercial trade occurs but trade is unorganized and institutional mechanism is poor.	On average >70% trees harvested annually for fruit. NTFP collection is carried out for commercial trade, and is organized through government supported institutions (VFPC & SHG) or through private traders
**Lopping of branches or stems for fruit harvest**	No lopped branches or stems of study species observed in plot. Harvesters confirm these are never cut for fruit harvest.	Lopped branches or stems of study species observed in plot. Harvesters confirm that stems and branches are sometimes cut for fruit harvest.	
**Cattle grazing**	No signs of livestock grazing and no cattle dung was observed. Harvester’s information corroborates that no livestock are grazed here. Villages with livestock are >5kms away from plot.	Cattle dung present on the way to the plot but not in the plot. Harvesters inform that sometimes grazing takes place. Nearest village with livestock < 5kms from plot.	Cattle dung present in the plot. Harvesters inform that site is a preferred grazing area. Nearest village with livestock is within 1km from plot.
**Fire frequency**	Harvesters report no history of fire incidences in the past 5 years and no signs of past fires.	Harvester report one fire incident in the past five years and there are signs of past fire	Harvesters report more than one incident of fire in the past five years and there are signs of recent fire
**Mistletoe** (*Taxillus tomentosa*) **infestation**	Mistletoe not observed on trees in the plot or in neighboring trees and harvesters confirm that it has not been sighted.	Mistletoe observed only on neighboring trees, but not on trees in the plots.	Mistletoes found on trees in the plots.

### Assessment of anthropogenic and environmental variables

In each plot we rated the levels of harvest, number of stems lopped for fruit harvest, cattle grazing, fire frequency and presence of mistletoe based on the triangulation of three sources of information: (i) quantitative evidence within the plot, (ii) quantitative evidence from forests surrounding the plot during monitoring and reconnaissance surveys, and (iii) information from harvesters (Table 2). Indigenous harvesters hold in-depth ecological knowledge of the forests [28, 29] and are excellent sources for detailed information on historical and recent levels and patterns of these anthropogenic disturbances.

We ranked the level of fruit harvest, as low, medium and high, based on the proportion of trees (number of harvested adult trees/number total adult trees) annually harvested, on average, for their fruit. This information was obtained from harvesters during the monitoring of the 1 ha plots and was consistent with information from trader sources on harvest from each region, and with observations by A.V. over 5 years. Given the time investment involved in locating and harvesting fruiting trees, trees are either harvested for all available fruit (approximately 90% of fruit crop) [27, 15] or not harvested at all. Harvesting intensity was fully consistent with whether harvest was for subsistence or commercial purposes, and with respect to the latter, whether there was an institutional mechanism for collection, storage, and sale of the fruit (Table 2). For example, when harvest was for subsistence, levels were always low and <10% of trees was harvested. When harvest was for commercial purposes and the different aspects of trade (collection, storage and sale) was organized, then harvest was consistently high (>70%). If commercial harvest was not organized, then harvest levels on average fell somewhere between 20–40% of trees (medium). There were no sites that fell in between categories.

We had no intermediate levels of lopping and so used only two categories, low and medium (Table 2). There was no secondary information available on the effects of grazing and fire on any of the three species, so we based our information on the knowledge of the harvesters who were also the cattle grazers in many cases. We categorized cattle grazing and fire frequency levels as high, medium, low (Table 2). Across the different sites it was consistently mentioned that the study species were not preferred fodder species, but we still consider this factor important since trampling by cattle may cause damage to the saplings or seedlings. Where mistletoe was present in our study plots, infection rates were similar, so we used one category for plots with mistletoe. There was no information available on the effects of disturbance on *T*. *chebula*, but since it shares a similar life-history to *P*.*emblica*, we used the same relative rankings as for *Phyllanthus spp*.

We also calculated the distance in kilometers to the nearest village using a GPS. This reflects, at least in part, the intensity of firewood harvest, and other sources of disturbance, such as increased trampling due to the volume of people passing through on their way to do other activities like forest produce harvest, grazing, etc.

We ranked environmental variables like canopy openness using visual estimates from the center of the plot. As this variable was highly correlated with forest type (with savanna woodlands having open canopies and all dry deciduous forests having more closed canopies), we used only forest type in the analyses. None of our other predictors were highly correlated (all pairwise correlations <0.6).

We based our rankings for the anthropogenic variables on the ecological information available from other research on these species, most of which comes from the nearby protected area, the Biligiri Rangaswamy Temple Wildlife Sanctuary (BRT). Our high harvest categories correspond with the level shown to decrease population growth rates of *Phyllanthus* spp. based on a ten-year population dynamics study in BRT [15]. Similarly, our high fire frequency category corresponds to the frequency at which growth rates of *P*. *emblica* populations have been shown to drop below unity [30]. Lopping large branches increases mortality in *Phyllanthus* spp. [27] and even small increases in adult mortality can decrease population growth rates [15]. Mistletoe infection on *Phyllanthus* spp. trees significantly decreases fruit production [31, 15]. For all variables, our relative rankings reflect mean levels over time and provide an appropriate approach for identifying predictors of population structure, which results from anthropogenic and environmental factors acting over multiple years.

### Data analyses

#### Characterizing population structure

The population structure of shade-tolerant, slow-growing forest trees that recruit regularly is typified by reverse J curves [32, 33]. For each study species within each plot, we characterized population structure by dividing stems into ~5 cm—wide size classes based on diameter at breast height (dbh), where size class 1 (seedlings) included stems <1 cm (regardless of height), size class 2 (saplings): 1 ≤ x < 5 cm, size class 3: 5 ≤ x < 10 cm, through size class 8 with stems ≥ 30 cm. Since seedling regeneration can be variable from year to year, we omitted individuals < 1 cm dbh from the model analyses to avoid confounding sampling seasons with regeneration rate. To assess the recruitment status of populations, we calculated the proportion of saplings (1–5 cm dbh), out of the total population. A greater proportion of saplings indicated a greater recruitment rate in the population. We also calculated the coefficient of skewness (*g*_1_), which provides a measure of the relative proportion of small (starting at sampling size) versus large individuals in a population and is a good predictor of the direction of population change (i.e. increase or decrease) in other tropical Asian tree species [17]. We calculated skewness as:

$$\text{-}g1=\frac{n\sum_{i}\left(xi\text{-}\bar{x}\right)\text{³}}{\left(n\text{-1}\right)\left(n\text{-}2\right)s\text{³}}$$

where *n* is the number of individuals; *x*_*i*_, is the dbh (in cms) at the midpoint of the size class of individual I;$\bar{x}$, the mean of *x*_*i*_ and *s*, is the standard deviation of *x*_*i*_. Populations with g_1_ > 0 have many larger individuals and relatively few small individuals and tend to decrease over time, while populations with g_1_ < 0 have many small individuals and fewer large individuals and tend to increase over time [17]. To ensure that estimates were not influenced by sample size, we scaled populations to 1,000 individuals. For this analysis, we used 10 cm dbh size classes, again omitting individuals < 1 cm dbh.

#### Evaluating the importance of anthropogenic and environmental factors

To determine what forms of anthropogenic disturbance and environmental factors affected population structure and recruitment, we used coefficient of skew and proportion saplings as response variables, respectively. We conducted the analyses for 23 *T*. *chebula* populations separately from the 31 populations of *P*. *emblica* and *P*. *indofischeri* because of potential differences in responses to disturbance and environmental factors between genera. Coefficient of skewness was modeled using multiple linear regression mixed-effects models [34]. The proportion of saplings was modeled using logistic regression mixed-effects models.

For all species we tested five measures of anthropogenic disturbance as predictors of population structure and recruitment status–intensity of fruit harvest, cattle grazing, presence or absence of lopping for fruit harvest, distance to nearest village, and fire frequency–as well as two environmental variables–altitude and forest type (savanna woodland vs. dry deciduous forest) (Table 2). For analyses of the *Phyllanthus* species, we additionally included levels of mistletoe infestation and species (*P*. *emblica* vs. *P*. *indofischeri*) as predictors. The forms of anthropogenic disturbance, as well as mistletoe infestation, were coded as 0, 1, or 2, corresponding with low, medium, and high intensities, described in Table 2. Forest type was treated as categorical. Distance to nearest village ranged from 1–10 kms. Altitude ranged from 715–1600 m, with a median of 1360 m. Since multiple villages are situated within locations (Table 1) and various plots may be located within a single village area, we considered village nested within location as random factors in all of our models. All analyses were conducted in the program R, version 2.13.1 [35].

In order to determine the impact and relative importance of multiple anthropogenic disturbances and environmental factors on population structure and levels of regeneration, we used an information-theoretic model-averaging approach [36]. Rather than selecting a single “best” model, this approach integrates information from all candidate models to assess the relative importance of the multiple anthropogenic and environmental factors and each factors’ effect on population regeneration status. We used all possible combinations of the seven (for *T*. *chebula*) or nine (*Phyllanthus* spp.) predictor variables in our model set [36]. We did not have sufficient replication of combinations of predictor variables to test for higher-order interactions. This yielded a total of 128 possible models for *T*. *chebula* and 512 possible models for *Phyllanthus* spp.

To assess the overall effect of each predictor variable on population structure and regeneration, we calculated AICc (second order Akaike information criteria, used for small sample size), differences in AICc, and AICc weights for each model in the two model sets (*T*. *chebula* and *Phyllanthus* spp.) using the R package AICcmodavg [36]. We calculated the mean and 95% confidence interval for the regression coefficient of each predictor variable by averaging coefficients across all models, weighted by the AICc weight of each model. When a 95% confidence interval for the regression coefficient of a predictor variable excluded zero, we interpreted this as a significant effect of the anthropogenic disturbance or environmental factors on population structure or regeneration. We also ranked the importance of the predictor variables according to the sum of AICc weights (ranging from 0 to 1) for models in which the variable was present. Because each predictor variable occurred in an equal number of candidate models, variables with higher sums of AICc weights were more important to explaining variation in the response variable (coefficient of skewness or proportion of saplings).

## Results

### Population structure

**Fig 2:**
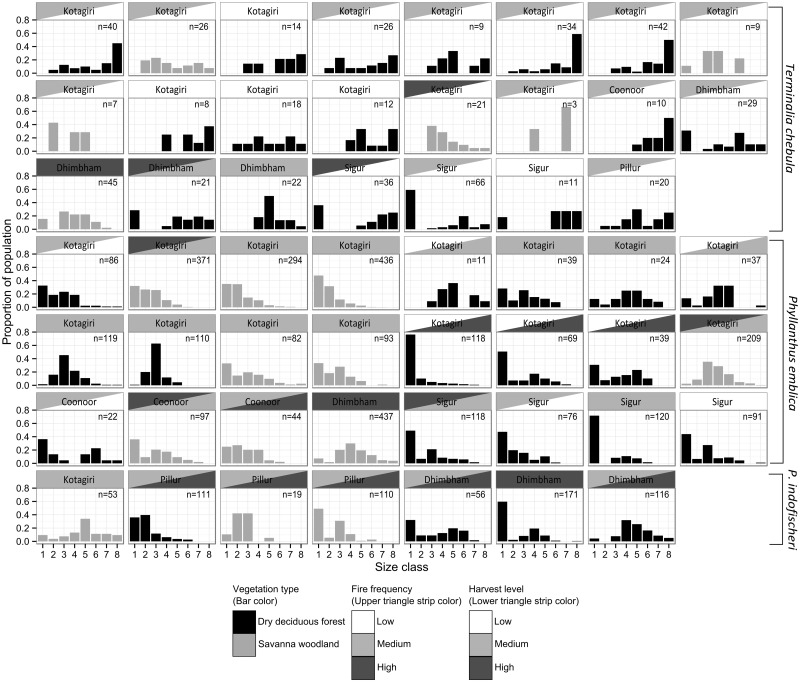
Size class distributions of populations of *Terminalia chebula*, *Phyllanthus emblica* and *Phyllanthus indofischeri* in the Nilgiri Biosphere Reserve. Size classes 1 through 8 represent 5cm dbh divisions, with size class 1 including individuals <1 cm dbh, size class 2 from 1–5 cm dbh, size class 3 from 5–10 cm dbh, through size class 8 with individuals greater than 30 cm dbh. Trees may become reproductive at ~ 5cm dbh for P. indifischeri and ~9–10 cm dbh for P. emblica and T. chebula. The region of the population is indicated above each graph. Dry deciduous forest populations are shaded black, while savanna woodland populations are shaded gray. Additionally the levels of harvest and fire disturbance for each population is indicated by coloring the strip text, with the upper triangle indicating fire frequency and the lower triangle indicating harvest levels.

Population structure varied across the three species and 41 plots (Fig. 2). None of the 23 *T*. *chebula* populations had a structure that suggested adequate proportion of seedlings or saplings. Instead populations tended to have distributions that were either dominated by adults or uniform. In over 80% (19/23) of the populations, the percent of seedlings (< 1 cm dbh) was ≤ 5. There were no seedlings in 60% of the populations, and 70% of the populations had no saplings (1–5 cm dbh).

In contrast, over two thirds of the *P*. *emblica* populations (17/24) and *P*. *indofischeri* populations (7/9) showed the typical reverse-J curve characteristic of regenerating populations of shade-tolerant forests trees with continuous recruitment. Almost all had *g*_1_ values that were negative, the two that did not, had values that were ~ 0. Only one *P*. *emblica* population had no seedlings and < 10% (2/24) had no saplings. Similarly only one *P*. *indofischeri* population had few to no abundance of seedlings and saplings.

Densities of trees ≥10 cm dbh in our study plots were variable, with means ± 1 SE of 16.7 ± 2.0, 39.6 ± 12.5 and 36.2 ± 12.0 trees/ha for *T*. *chebula*, *P*. *emblica*, and *P*. *indofischeri* respectively. We also found that trees may become reproductive at ~5 cm dbh for *P*. *indifischeri* and ~ 9–10 cm dbh for *P*. *emblica* and *T*. *chebula*.

### Predictors of recruitment status

Vegetation type was the most important predictor of the proportion of saplings in *T*. *chebula* populations, with a significantly higher proportion of saplings found in savanna woodland than in dry deciduous forest, as shown by the 95% confidence interval for the model-averaged estimated effect (Table 3a). Populations where branches or stems were lopped for harvest also had a significantly higher proportion of saplings. In contrast, areas with a high fruit harvest intensity and those with high fire frequency had significantly lower proportions of saplings.

The only significant predictor of the proportion of *Phyllanthus* spp. saplings was vegetation type, with significantly more saplings in savanna-woodland plots than in dry forest plots as shown by the 95% confidence interval for the model-averaged estimated effect (Table 3b). Although our plots spanned the range of fruit harvest intensities, for *Phyllanthus* spp. fruit harvest alone was a poor predictor of recruitment.

**Table 3:** Model-averaged regression coefficients for all variables included as predictors of proportion of saplings for a) *T. chebula* and b) *Phyllanthus* spp. populations, ranked in descending importance by the sum of AICc weights of candidate models including the variable under consideration.

Variable	Rank	Sum of AICc weights	Model-averaged estimate	Standard error	95% confidence interval for estimate
***a) T*. *chebula***					
**Vegetation type (savanna woodland)**	**1**	**0.974**	**2.5241**	**0.7957**	**0.9644, 4.0837**
**Cut stems**	**2**	**0.639**	**1.4768**	**0.7017**	**0.1015, 2.8522**
**Harvest**	**3**	**0.515**	**-1.4128**	**0.6985**	**-2.7819,-0.0437**
**Fire**	**4**	**0.409**	**-1.2469**	**0.6293**	**-2.4803,-0.0135**
Distance	5	0.397	-0.2601	0.1618	-0.5773, 0.057
Altitude	6	0.263	-0.002722	0.002012	-0.006665, 0.001221
Grazing	7	0.145	-0.3553	0.5098	-1.3545, 0.644
**b) *Phyllanthus* spp.**					
**Vegetation type (savanna woodland)**	**1**	**0.615**	**0.5228**	**0.259**	**0.0151, 1.0305**
Fire	2	0.498	-0.2625	0.1568	-0.5699, 0.0449
Grazing	3	0.446	0.3601	0.2371	-0.1045, 0.8248
Altitude	4	0.396	-0.000839	0.000623	-0.00206, 0.000382
Distance	5	0.349	0.0911	0.084	-0.0735, 0.2556
Harvest	6	0.244	0.1525	0.3615	-0.5561, 0.861
Species (indofischeri)	7	0.209	-0.5505	0.7025	-1.9274, 0.8263
Parasite	8	0.2	-0.2396	0.3911	-1.0062, 0.5269
Cut stems	9	0.197	0.0619	0.1606	-0.2529, 0.3767

Variables in bold have model-averaged estimates whose 95% confidence intervals exclude zero.

For *T*. *chebula*, none of our measures of anthropogenic disturbance (Table 2) explained a substantial amount of the variation in the coefficient of skewness across populations (Table 4a). Based on AICc weights and confidence intervals for regression coefficients, altitude appears to be the only variables important to explaining differences in coefficient of skewness. Populations at higher elevations have more individuals in small size classes, and therefore a more negative coefficient of skewness.

**Table 4:** Model-averaged regression coefficients for all variables included as predictors of coefficient of skewness for a) *T. chebula* and b) *Phyllanthus* spp. populations, ranked in descending importance by the sum of AICc weights of candidate models including the variable under consideration.

Variable	Rank	Sum of AICc weights	Model-averaged estimate	Standard error	95% confidence interval for estimate
**a) *T. chebula***					
**Altitude**	**1**	**0.936**	**-0.0017**	**0.0005**	**-0.0028,-0.0007**
Distance to village	2	0.244	0.0723	0.058	-0.0414, 0.1861
Harvest	3	0.164	-0.1893	0.2224	-0.6252, 0.2467
Vegetation type (savanna woodland)	4	0.127	0.0609	0.2831	-0.494, 0.6158
Grazing	5	0.125	-0.0599	0.149	-0.352, 0.2323
Fire	6	0.12	-0.0022	0.1811	-0.3573, 0.3528
Cut stems	7	0.118	-0.008	0.2226	-0.4442, 0.4283
**b) *Phyllanthus* spp.**					
**Vegetation type (savanna woodland)**	**1**	**0.895**	**-0.8034**	**0.2636**	**-1.3201,-0.2868**
**Altitude**	**2**	**0.603**	**0.0013**	**0.0006**	**0.0001, 0.0024**
**Fire**	**3**	**0.575**	**0.3539**	**0.1664**	**0.0277, 0.6802**
Parasite	4	0.19	0.1926	0.2996	-0.3946, 0.7797
Cut stems	5	0.175	0.166	0.2267	-0.2784, 0.6103
Species (indofischeri)	6	0.162	-0.1374	0.4561	-1.0313, 0.7564
Distance	7	0.16	-0.0061	0.0697	-0.1427, 0.1306
Harvest	8	0.159	0.0989	0.2168	-0.326, 0.5238
Grazing	9	0.151	-0.0213	0.1839	-0.3816, 0.3391

Variables in bold have model-averaged estimates whose 95% confidence intervals exclude zero. A positive estimated effect indicates the predictor variable is associated with more large individuals and fewer small individuals.

Plots with higher frequency of fire were significantly associated with more positive coefficients of skewness for *Phyllanthus* spp. populations, indicating that populations in these conditions had relatively few small individuals (Table 4b). Populations in savanna woodlands and those at higher elevations were significantly associated with more negative coefficients of skewness, as they had more small individuals and fewer large individuals compared to populations in dry deciduous forest. Plots with high levels of mistletoe infection had higher coefficients of skew, although the 95% confidence intervals for estimates of the effect of these factors include zero. Harvest intensity was not a significant predictor of the coefficient of skew for *Phllyanthus* spp.

## Discussion

### Effects of fruit harvest on regeneration

Our results demonstrate limitations in the recruitment of *T*. *chebula* populations across the Nilgiri Biosphere Reserve (NBR). Although population structure does not necessarily predict dynamics, in slow-growing, shade-tolerant species with continuous recruitment like *T*. *chebula*, no/very low recruitment through to the sapling stage is a good indication of a problem [32]. These findings are consistent with population surveys elsewhere in the Western Ghats [14, 37] that have reported very low levels of recruitment for this species. Our results suggest that high levels of fruit harvest, in addition to frequent fire are the main contributors to low regeneration.

While most *P*. *emblica* and *P*. *indofischeri* populations appear to be recruiting well, our results suggest that one third of them had poor recruitment. Studies of *P*. *emblica* at various other sites in the Western Ghats found reduced levels of recruitment in sites closer to human settlements and with higher human disturbance and assumed the major cause was fruit harvest [39, 13, 14]. In contrast, our results show that neither fruit harvest, nor distance to nearest village are significant predictors of *Phyllanthus spp* recruitment, even though our plots spanned the full range of fruit harvest intensities (e.g. from no/low to high fruit harvest). Scoles & Gribel [20] also found that fruit harvest was not a significant predictor of recruitment of brazil nut trees, and various studies have illustrated that some species of trees may withstand very high levels of fruit harvest over the long-term (up to 80–90%) without population decline [8]. This is because reductions in fecundity for woody species tend to have little effect on long-term population growth rates [39]. In a demographic study of *Phyllanthus* spp. in a nearby protected area, Ticktin et al. [15] showed that long-term fruit harvest at the level considered “high” here significantly decreases population growth rates, but does not push them below replacement levels, unless populations are simultaneously subject to the effects of mistletoe or invasive species. Those study populations were not subject to frequent fire.

The fact that distance to village was not significantly correlated with harvest intensity or any of our measures of anthropogenic disturbance (the closest relationship was a tendency for plots closer to villages to have more frequent fires, r = 0.3, p<0.1), suggests that the common use of distance to nearest village as a proxy for disturbance level may not always be appropriate. Although harvesters here confirmed firewood extraction varies as a function of distance from village, firewood collection involves only removal of twigs and dead branches but not of live material, and there is no commercial sale. This relatively low pressure, and the fact that our study species are not preferred firewood species, may explain why we did not detect an effect on recruitment levels in the population structure.

### Other anthropogenic drivers of NTFP regeneration

Our results suggest that other factors are also important predictors of recruitment of our study species. Our models illustrate that frequent fire is significantly correlated with reduced proportions of *T*. *chebula* saplings, and with a positive coefficient of skewness (fewer saplings) in *Phyllanthus* spp. The effects of fire on regeneration of dry forest plants vary among species and depend on fire intensity and frequency [41, 42, 19]. In the NBR fires are almost always surface fires and mean fire return intervals for dry forests is six years [19]. Although fire has been used as a management practice in the Western Ghats for over 3,500 years [11], and the study species can resprout after fire and may benefit from the reduction in cover of other understory species, the frequency and spatial extent of fire in dry deciduous forests of the NBR has increased in recent decades [19]. Increasing frequencies of fire have been shown to significantly reduce densities of dry forest seedlings and saplings in the NBR and elsewhere in South India [43,19]. Other research has shown specifically that *P*. *emblica* populations decrease with increasing frequency of high intensity fires [30].

We note that more homogenous size-class distributions do not necessarily indicate lower regeneration, as they can also be a result of faster growth in small size classes and/or a higher survival rate [44]. However, this is not likely the case in our study, since individuals of our study species are slow growing and fire decreases rates of survival and growth (due to continual resprouting) of saplings.

The significant increase in the proportion of *T*. *chebula* saplings in plots where branches or stems are lopped to obtain the fruit most likely does not indicate increased recruitment, but a decreased number of adult plants. This may be due to increased mortality of trees from cutting stems at the base or lopping branches. If so, this is expected to have negative impacts on long- term population growth rates, as the dynamics of long-lived, slow growing species tend to be most sensitive to changes in adult mortality [40]. Elsewhere in the Western Ghats, Sinha & Bawa [31] showed that lopping of *Phyllanthus* spp. branches to obtain fruit, significantly reduces fruit production the following year, and hypothesized that it can also reduce adult survival. The fact that we found no effect of lopping on *Phyllanthus* spp. population structure may be due to the relatively low levels observed: in plots where lopping was practiced, 5–10% of the trees were affected but the great majority of lopping involved cutting branches high up in the tree. In Sinha & Bawa’s study, 15% of *P*. *indofischeri* trees were lopped at the base. At higher intensities, lopping of branches and stems could have impacts similar to selective logging, which can be a major cause of decline for tree species with both timber and NTFP uses [45, 47].

Grazing pressure was not a significant predictor of population structure for either species, based on the proportion of saplings in a population or the coefficient of skew. Although cattle grazing can reduce the proportion of saplings in a population [48], the reverse can occur if individuals in grazed areas remain saplings for long periods due to continual grazing and resprouting [49]. However, our estimates of grazing pressure were coarse and further research that quantifies rates of grazing across years on population dynamics of this species is necessary to evaluate these possibilities.

### Effects of environmental factors on regeneration

For both *T*. *chebula* and *Phyllanthus* spp., environmental variables were more important predictors of recruitment than any of the anthropogenic disturbance types we quantified. The more negative coefficients of skew (relatively more smaller individuals) in both genera at higher elevation appears to be an artifact of the differences in the vegetation structure between these environments: plots at higher altitudes can support lower stature vegetation and so lack the large individuals found at lower altitudes. However, the significantly higher proportions of saplings of both genera in savanna woodlands than in dry forests is likely attributed to the more open canopies in the former (vegetation type was significantly correlated with canopy openness, r = 0.79, p<0.01). The higher light likely allows for the higher regeneration and recruitment. Since there were no differences in intensity of fruit harvest (or other anthropogenic factors) between these two habitat types, these results suggest that populations in savanna woodlands may be able to withstand higher rates of fruit harvest and other types of disturbance that may lower regeneration than those in dry forests. This is consistent with other studies of other NTFP species, where populations in higher light environments such as secondary forests are more resilient to harvest than those in old growth environments [50, 51].

### Additional potential factors affecting regeneration status

The regeneration patterns that we documented are likely also a result of other, confounding factors. For example, Shanker et al. [14] found low levels of genetic diversity for *T*. *chebula* and hypothesized that this may be related to low observed levels of regeneration. Shaanker et al. [26] argued that poor regeneration in *T*. *chebula* could also be perhaps be a result of high level of developmental lethals in the system, which could in turn be a possible consequence of high levels of inbreeding in reduced populations. In addition, germination rates of *T*. *chebula* in nurseries are very low (A.V. unpublished data) and a high proportion (~50%) of seeds on the forest floor lack embryos (A.V. pers. obs.). *T*. *chebula* is bee pollinated and pollinator deficiency has been proposed as a possible contributor to low regeneration for this species elsewhere in the Western Ghats [38]. The role of these additional factors needs further investigation.

Insufficient seed dispersal may also contribute to low regeneration for these species. [38]. Seeds of all three species are dispersed by mammals, and although hunting has been illegal since the Wildlife Protection Act of 1972, seed disperser populations may have decreased due to habitat loss, fragmentation and other reasons.

For *Phyllanthus* spp. populations in the nearby Biligiri Rangaswamy Temple Wildlife Sanctuary (BRT), mistletoe has been shown to be a major driver of population decline [15]. In our study plots the number of trees infected by mistletoes was much lower than in the BRT, where it is common to find 90% of adult trees in a stand infected. This likely explains why we found only a tendency for plots with high levels of mistletoe to have a lower proportion of saplings. However, if mistletoes were to increase in the NBR, this could also decrease recruitment.

### Implications for forest management and conservation

Many NTFP populations in the tropics are subject to multiple stressors. Our results illustrate the importance of identifying which ones, in addition to harvest; have the greatest negative effects on recruitment. This is not at all obvious. For example, although fruit harvest has been blamed for observed declines in our three species and federal policy implemented to restrict harvest in protected areas, our results suggest that prohibiting fruit harvest alone is likely ineffective conservation policy and will not improve the status of these populations. Ineffective conservation policy for NTFPs not only fails to protect species but also can have large negative economic repercussions for harvesters [12].

To maintain *T*. *chebula* and *Phyllanthus* spp. populations as well as those of other NTFPs, conservation policies and education must be directed to reducing the various disturbance factors identified here. For example, participatory monitoring [28] of fire frequency could help to determine areas that are vulnerable to frequent fires and the reasons why. Steps could then be taken to devise management plans to decrease fire frequency and to decrease the practice of lopping branches and stems. This kind of participatory monitoring and adaptive management holds great potential under India’s new Forest Rights Act, which restores indigenous rights to harvest forest resources and manage customary areas, and stipulates sustainable use and co-management with the Forest Department [52]. Fruit is not currently harvested from *T*. *chebula* due to decreased demand, but should it increase again, effective limits could be promoted by alternating the harvest of populations over space (populations within a village) and time. For all of the three study species, monitoring to ensure regeneration is occurring is important. Although mistletoe infection is not currently intense or widespread in the NBR, methods for reducing mistletoe infestations have already been evaluated [53], and can be promoted and tested over the longer term by harvesters.

One complication of identifying and addressing causes of decline is that different kinds of disturbance are likely related. For example, Goldammer [46] suggests that logging, grazing and NTFP harvest may affect spatial and temporal patterns of fire in dry forests. Similarly, the mistletoes that infest *Phyllanthus* spp. populations are native species that have likely spread as a result of changes in land management, including fire management [54]. Although in some South Indian protected areas regeneration of dry forest species, including *Phyllanthus* spp. is severely limited by the introduced invasive species *Lantana camara* L. [37, 14], lantana and other invasive species were uncommon in our forest plots. This in turn could be a result of the low intensity fires, which can control lantana [54] and are banned in protected areas but still persist in the NBR’s reserve forests. These interrelationships among different types of anthropogenic and environmental disturbances point to the complexity of–and great need for–gaining a better understanding of the multiple use of forests by local communities for designing effective forest management policy.

Recent studies have emphasized the need for incorporating drivers into models of the population dynamics of species of conservation concern [55, 6]. We have taken the first step towards doing this by assessing population structure to identify which of many possible drivers may be most important to consider. The use of a multi-model inference-based approach allowed us to determine which subset of factors should be the focus of further investigation. One reason that this has rarely been done in the tropics is the difficulty in obtaining information on current and historical levels of drivers. We used a participatory approach to involve local communities in the research and triangulate their knowledge of historic and recent disturbance with field observations. As such, we were able to obtain robust rankings of the types of anthropogenic disturbance we assessed. Further research to assess the effects of these anthropogenic drivers on the population dynamics of these and other species in multiple-use environments is now needed to provide clearer insight on best forest management and conservation strategies.
